# The novel zebrafish model *pretzel* demonstrates a central role for SH3PXD2B in defective collagen remodelling and fibrosis in Frank-Ter Haar syndrome

**DOI:** 10.1242/bio.054270

**Published:** 2020-12-29

**Authors:** Ivo J. H. M. de Vos, Arnette Shi Wei Wong, Jason Taslim, Sheena Li Ming Ong, Nicole C. Syder, Julian L. Goggi, Thomas J. Carney, Maurice A. M. van Steensel

**Affiliations:** 1 Skin Research Institute of Singapore (SRIS), Agency for Science, Technology and Research (A*STAR), 308232, Singapore; 2 Institute of Medical Biology (IMB), Agency for Science, Technology and Research (A*STAR), 138648, Singapore; 3 Singapore Bioimaging Consortium (SBIC), Agency for Science, Technology and Research (A*STAR), 138667, Singapore; 4 Department of Physiology, Yong Loo Lin School of Medicine, National University of Singapore (NUS), 117593, Singapore; 5 Lee Kong Chian School of Medicine, Nanyang Technological University (NTU), 636921, Singapore

**Keywords:** FTHS, SH3PXD2B, ECM remodelling, Fibrosis

## Abstract

Frank-Ter Haar syndrome (FTHS, MIM #249420) is a rare skeletal dysplasia within the defective collagen remodelling spectrum (DECORS), which is characterised by craniofacial abnormalities, skeletal malformations and fibrotic soft tissues changes including dermal fibrosis and joint contractures. FTHS is caused by homozygous or compound heterozygous loss-of-function mutation or deletion of *SH3PXD2B* (Src homology 3 and Phox homology domain-containing protein 2B; MIM #613293). *SH3PXD2B* encodes an adaptor protein with the same name, which is required for full functionality of podosomes, specialised membrane structures involved in extracellular matrix (ECM) remodelling. The pathogenesis of DECORS is still incompletely understood and, as a result, therapeutic options are limited. We previously generated an *mmp14a/b* knockout zebrafish and demonstrated that it primarily mimics the DECORS-related bone abnormalities. Here, we present a novel *sh3pxd2b* mutant zebrafish, *pretzel*, which primarily reflects the DECORS-related dermal fibrosis and contractures. In addition to relatively mild skeletal abnormalities, *pretzel* mutants develop dermal and musculoskeletal fibrosis, contraction of which seems to underlie grotesque deformations that include kyphoscoliosis, abdominal constriction and lateral folding. The discrepancy in phenotypes between *mmp14a/b* and *sh3pxd2b* mutants suggests that in fish, as opposed to humans, there are differences in spatiotemporal dependence of ECM remodelling on either *sh3pxd2b* or *mmp14a/b*. The *pretzel* model presented here can be used to further delineate the underlying mechanism of the fibrosis observed in DECORS, as well as screening and subsequent development of novel drugs targeting DECORS-related fibrosis.

This paper has an associated First Person interview with the first author of the article.

## Abstract (precis)

**Summary:** We report a novel *sh3pxd2b* mutant zebrafish model that develops cutaneous and musculoskeletal fibrosis, similar to that observed in people with Frank-Ter Haar syndrome.

## INTRODUCTION

Frank-Ter Haar syndrome (FTHS, MIM #249420) is a skeletal dysplasia, characterised by craniofacial abnormalities, skeletal malformations, and a generalised reduction in bone mineral density. Eye, heart, and skin are also commonly affected (Bendon et al., 2012; Borrone et al., 1993; Chang et al., 2017; Hamel et al., 1995; Iqbal et al., 2010; Maas et al., 2004; Mégarbané et al., 1997; Ratukondla et al., 2020; Ter Haar et al., 1982; Wilson et al., 2014; Zrhidri et al., 2017). FTHS is inherited as an autosomal recessive trait, caused by homozygous or compound heterozygous loss-of-function mutation or deletion of *SH3PXD2B* [Src homology 3 (SH3) and Phox homology (PX) domain-containing protein 2B; MIM #613293] located at 5q35.1 (Buschman et al., 2009; Iqbal et al., 2010). The disorder is rare – to date, only 22 patients with a molecularly confirmed diagnosis have been reported (Bendon et al., 2012; Borrone et al., 1993; Chang et al., 2017; Hamel et al., 1995; Iqbal et al., 2010; Maas et al., 2004; Mégarbané et al., 1997; Ratukondla et al., 2020; Ter Haar et al., 1982; Wilson et al., 2014; Zrhidri et al., 2017). *SH3PXD2B* encodes an adaptor protein with the same name, alternatively known as tyrosine kinase substrate with four SH3 domains (TKS4), with an amino (N-) PX domain that binds phosphoinositides [PI3P and PI(3,4)P_2_] in membranes, and four SH3 domains that bind other proteins (Buschman et al., 2009; Iqbal et al., 2010). It is involved in recruiting matrix metalloproteinase 14 (MMP14) to membrane structures called podosomes, which adhere to and degrade extracellular matrix (ECM) (Buschman et al., 2009; Murphy and Courtneidge, 2011; Hoshino et al., 2013). Via its link with MMP14 at podosomes, SH3PXD2B facilitates ECM remodelling and invasive cell motility (Buschman et al., 2009). We recently reviewed *SH3PXD2B*, *MMP14* and *MMP2* related syndromes, and concluded that these disorders form a spectrum in which defective collagen remodelling plays a central role. As such, we proposed to refer to this spectrum as defective collagen remodelling spectrum (DECORS) (De Vos et al., 2019). The pathogenesis of DECORS is still incompletely understood and, as a result, therapeutic options are limited. Earlier mouse models have primarily focussed on the SH3PXD2B and MMP14-related bone and eye phenotypes (Curtain and Donahue, 2007; Du et al., 2013; Dülk et al., 2016; Holmbeck et al., 1999; Iqbal et al., 2010; Mao et al., 2009; Mao et al., 2011; Vas et al., 2019; Zhou et al., 2000). However, mouse models are impractical for large-scale drug screening. A model animal more suitable for such endeavours would be the zebrafish (*Danio rerio*). We previously generated an *mmp14a/b* knockout (KO) zebrafish and demonstrated that it primarily mimics the DECORS-related bone abnormalities (De Vos et al., 2018, 2019). Here, we present a novel *sh3pxd2b* mutant zebrafish model that we named *pretzel* for its most striking feature. In contrast to our *mmp14a/b* KO model, *pretzel* primarily reflects the DECORS-related dermal fibrosis and contractures, which makes this fish a potentially promising model to support the development of anti-fibrosis therapy for DECORS patients.

## RESULTS

### Genome editing of *sh3pxd2b* in zebrafish

Most reported human, as well as murine, *SH3PXD2B* mutations result in protein truncation (Bendon et al., 2012; Chang et al., 2017; Iqbal et al., 2010; Ratukondla et al., 2020; Wilson et al., 2014; Zrhidri et al., 2017). In several patients, as well as in the *Nee* (nose, eyes, ear) mouse model, this truncation is located after the second SH3 domain (Bendon et al., 2012; Iqbal et al., 2010; Mao et al., 2009, 2011; Zrhidri et al., 2017). Zebrafish *sh3pxd2b* is predicted to encompass 14 coding exons, together encoding an 880 a.a. protein. Using CRISPR/Cas9, we edited the genomic sequence of *D. rerio sh3pxd2b* (Fig. S1A), resulting in a four-base pair (bp) deletion in exon 13 (Fig. S1B). This deletion was predicted to result in the truncation p.I375Mfs16X, keeping only the N-terminal 43% of the protein intact (Fig. S1C). Quantitative polymerase chain reaction (qPCR) analysis of 2-month-old *sh3pxd2b*^Δ/Δ^ homozygous mutants and their *sh3pxd2b*^+/+^ wild type (WT) clutch mates demonstrated that the CRISPR-induced mutation had no significant effect on *sh3pxd2b* mRNA expression (Fig. S1D; 8% increase in expression of mutant *sh3pxd2b* relative to WT *sh3pxd2b*, P=0.0706). Next, we assessed the effect of our CRISPR-induced mutation at the protein level. To our knowledge, there are currently no primary antibodies available that are validated for detection of zebrafish Sh3pxd2b. To circumvent this problem, we isolated WT and mutant zebrafish cDNA, cloned them into expression vectors where the *sh3pxd2b* coding sequence was flanked by a 5′ EGFP and 3′ HA coding sequence, and expressed the tagged WT or mutant fusion protein, respectively, in a relevant cell line (Fig. S1E) (De Vos et al., 2018). Due to difficulties with amplifying the *sh3pxd2b* coding sequence from zebrafish cDNA, we were only able to isolate the last two exons from WT and mutant cDNA, encompassing the CRISPR target site. Whole cell extract was subsequently subjected to immunoblotting with anti-GFP and anti-HA antibodies. The WT tagged protein was expected to show an 81 kDa band for both GFP and HA, while the mutant construct should only show a 31 kDA band for GFP. As expected, the mutant construct only showed a single band for GFP around 30 kDa (Fig. S1F, open arrow head). The WT construct did not show a band for HA either, whereas our previously published positive control showed a strong HA band at the expected height (Fig. S1F, solid and open arrow) (De Vos et al., 2018). Interestingly, the WT construct appeared as multiple GFP bands, all smaller than 81 kDa (Fig. S1F, solid arrow heads), suggesting that the WT construct was being cleaved. This unexpected cleavage might result from processing that differs from that in zebrafish cells, in addition to possible erroneous folding of the zebrafish Sh3pxd2b carboxyl (C-) terminus at 37°C (versus its normal 28.5°C). Regardless of this unexpected cleavage, all WT bands are larger than the mutant band, suggesting that we successfully generated a mutation in zebrafish *sh3pxd2b* with a similar truncating effect as mutations identified in FTHS patients.

### A novel zebrafish model for *SH3PXD2B* related DECORS, *pretzel*

**Fig. 1.**
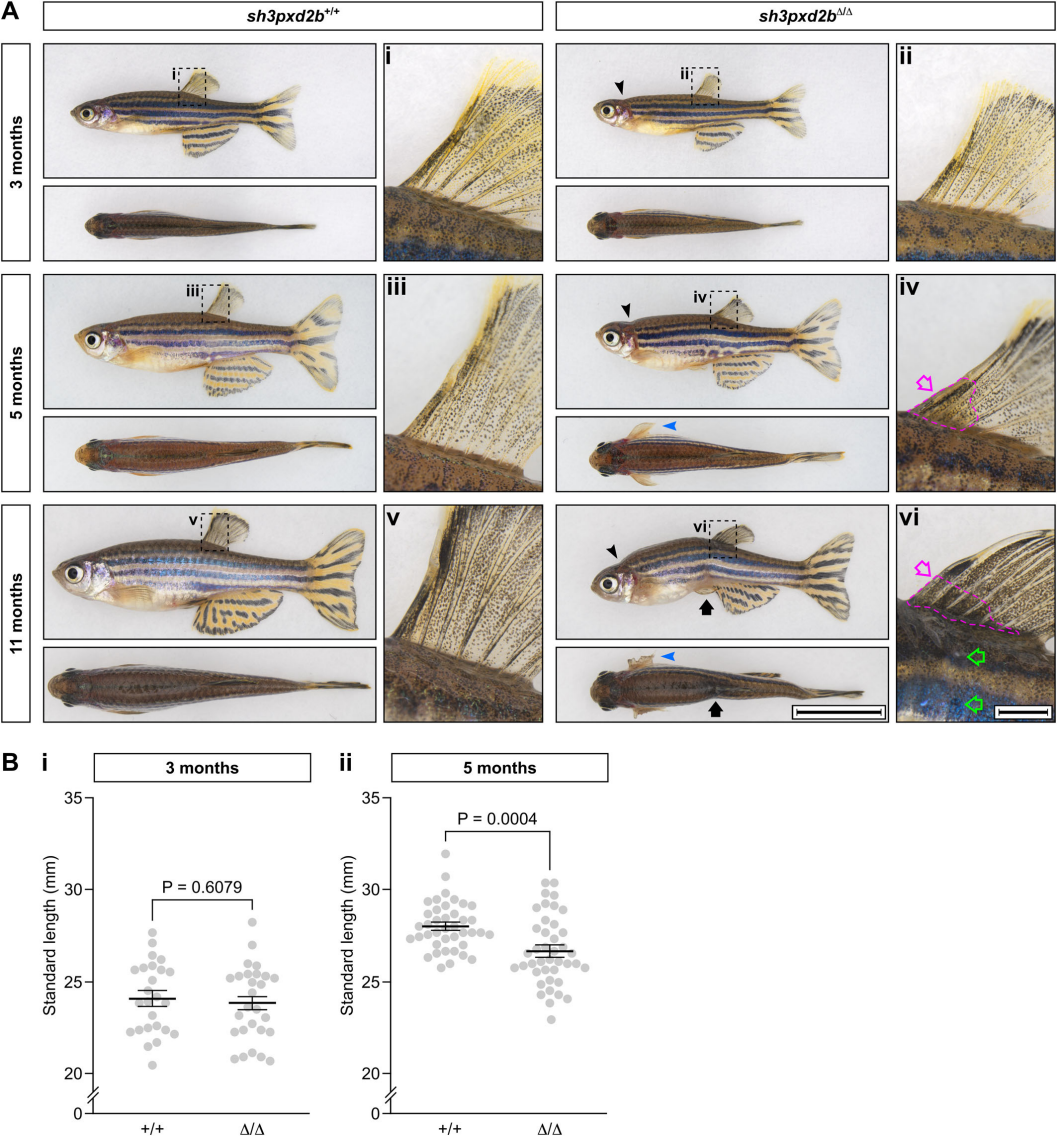
**Adult *sh3pxd2b*^Δ/Δ^ zebrafish have a gradually worsening phenotype, affecting skeletal structures and skin.** (A) Representative images of *sh3pxd2b*^Δ/Δ^ mutant fish and their *sh3pxd2b*^+/+^ WT clutch mates at the ages of 3, 5 and 11 months, selected from a total of 44 analysed fish per genotype per time point (see Table 1), imaged with a Nikon SMZ25 stereomicroscope. At 3 months of age, mutants demonstrate a short, up-tilted head (black arrowhead) with short operculum, exposing the gills (visible as a red patch caudal to the operculum); these features are absent in their WT clutch mates. At the age of 5 months, the pectoral fins of mutants are stiff and constantly kept in abducted position (blue arrowhead). At the anterior base of their dorsal fin, an opaque spot can be observed (iv, magenta arrow) that is absent in younger mutants and WT fish (compare with i–iii). At 11 months of age, mutants demonstrate hyper-kyphosis and abdominal constriction (black arrow). The pectoral fins of mutants are stiff and show signs of physical damage in multiple individuals (blue arrowhead). The opaque spot at the base of the dorsal fin (vi, magenta outlined arrow) is larger at 11 months of age and is hyper-pigmented (compare with iv and v). Note the sharper angle of fin rays with the dorsum of the fish in mutants (iv,vi) as compared to WT clutch mates (iii,v), as a result of the inability of the fins to be abducted manually. The flank, ventral to the dorsal fin, shows a whitish discoloration (green outlined arrows) in mutants. Scale bar equals 10 mm in overview and 1 mm in dorsal fin close-up. (B) Standard length measurements of 25 WT and 27 mutant 3-month-old fish (i), and 40 WT and 41 mutant 5-month-old fish. While there is no statistically significant difference in mean standard length between mutants and WT clutch mates at 3 months of age (i), at the age of 5 months mutants are significantly shorter than their WT clutch mates (*P*=0.6079 and *P*=0.0004, respectively, as assessed by two-sampled, non-pooled, two-tailed Student's *t*-test). Error bars represent standard error of means of biological replicates.

After establishing that we induced an *sh3pxd2b* mutation in zebrafish by CRISPR/Cas9 and conducting *in vitro* analyses that suggested truncation of the resulting protein product, we next assessed the phenotype of *sh3pxd2b*^Δ/Δ^ fish. Until young adult age (3 months post fertilisation), only the cranium of homozygous mutants seemed affected, as *sh3pxd2b*^Δ/Δ^ fish had a short, slightly up-tilted head (Fig. 1A, black arrow head), with a short operculum that exposed the underlying gills (see also Table 1 for prevalence of phenotypic features at different time points). At 3 months of age, there was no statistically significant difference in standard length (SL) between mutants (23.8 mm) and their WT clutch mates (24.1 mm, *P*=0.6079; Fig. 1Bi). As such, the phenotype of *sh3pxd2b*^Δ/Δ^ fish seemed similar, but milder than that of *mmp14a/b* KO fish (De Vos et al., 2018). From 5 months of age however, the phenotype of *sh3pxd2b*^Δ/Δ^ fish became more pronounced. The pectoral fins of mutants were usually kept in an abducted position (blue arrowhead) and could not be adducted manually. In addition, we noticed an opaque spot at the anterior-most base of the dorsal fin (Fig. 1Aiv, magenta arrow and outline). Furthermore, the mutants had a mild, yet statistically significantly shorter SL (26.7 mm) than their WT clutch mates (28.0 mm, *P*=0.0004; Fig. 1Bii). Six months later, at the age of 11 months, the pectoral fins were stiff and showed signs of physical damage in multiple mutants (Fig. 1A, blue arrowhead). The opaque dorsal fin spot had increased in size and was hyper-pigmented (Fig. 1Avi, magenta arrow and outline). The flank, ventral to the dorsal fin, had a whitish discoloration (green outlined arrows). At the age of 11 months, mutants showed abdominal constriction ventral to this discoloration, in addition to pronounced kyphosis (Fig. 1A, black arrow). As indicated in Table 1, none of these features were present in WT fish (with exception of a mild cranial up-tilt in a single individual). Due to the increasingly bent appearance of the *sh3pxd2b*^Δ/Δ^ fish over time, we decided to name this novel FTHS zebrafish model *pretzel*.

**Table 1.**
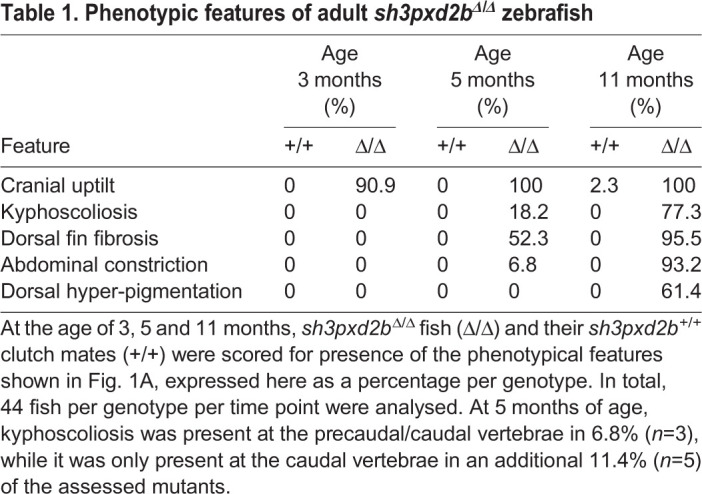
**Phenotypic features of adult *sh3pxd2b^Δ/Δ^* zebrafish**

### The *pretzel* zebrafish model develops dermal fibrosis at adult age

**Fig. 2.**
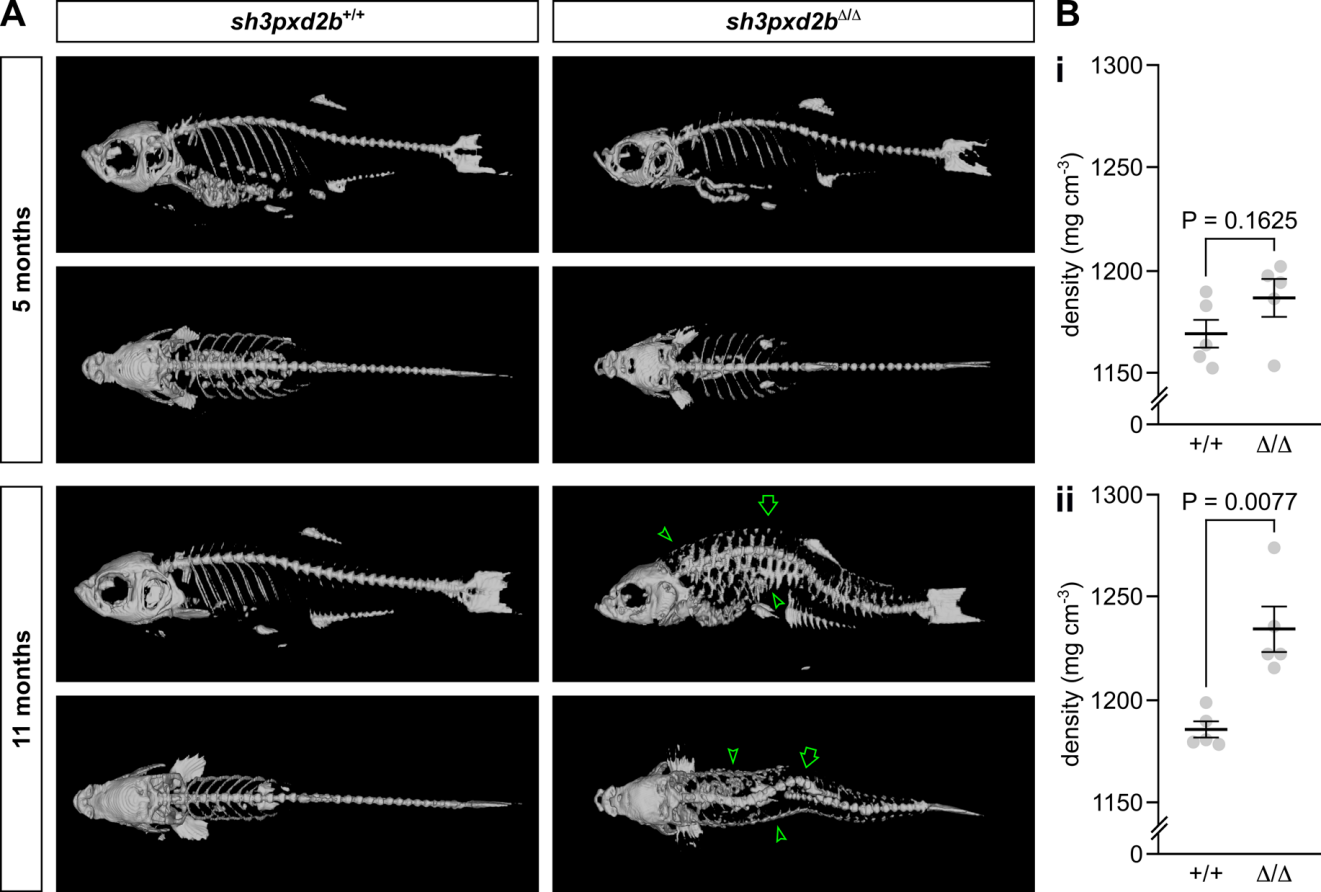
**Adult *sh3pxd2b*^Δ/Δ^ zebrafish develop kyphoscoliosis and an increased cutaneous density.** (A) Representative µCT 3D renders of *sh3pxd2b*^Δ/Δ^ mutant fish and *sh3pxd2b*^+/+^ WT clutch mates at the age of 5 and 11 months, selected form a total of five assessed fish per genotype per time point, imaged with a Siemens Inveon CT. At 5 months of age, mutant fish have a relatively small, up-tilted skull as compared to their WT clutch mates. At 11 months, mutants have developed kyphoscoliosis (outlined arrow). The skin of mutants shows an increased density (outlined arrow heads), that is not present at 5 months of age. Note that the fish displayed here had all the phenotypical features listed in Table 1 (see Fig. S2 for gross anatomy images). (B) Tissue density measurements of the five scanned fish per genotype per time point. At 5 months of age (i), there is a slight increase in overall tissue density of mutant (Δ/Δ) fish compared to their WT (+/+) clutch mates, which is however not statistically significant (NS; *P*=0.1625). At 11 months of age (ii), the overall tissue density of mutant fish is significantly increased compared to their WT clutch mates (*P*=0.0077; assessed by two-sampled, non-pooled, two-tailed Student's *t*-test). Error bars represent standard error of means of biological replicates.

**Fig. 3.**
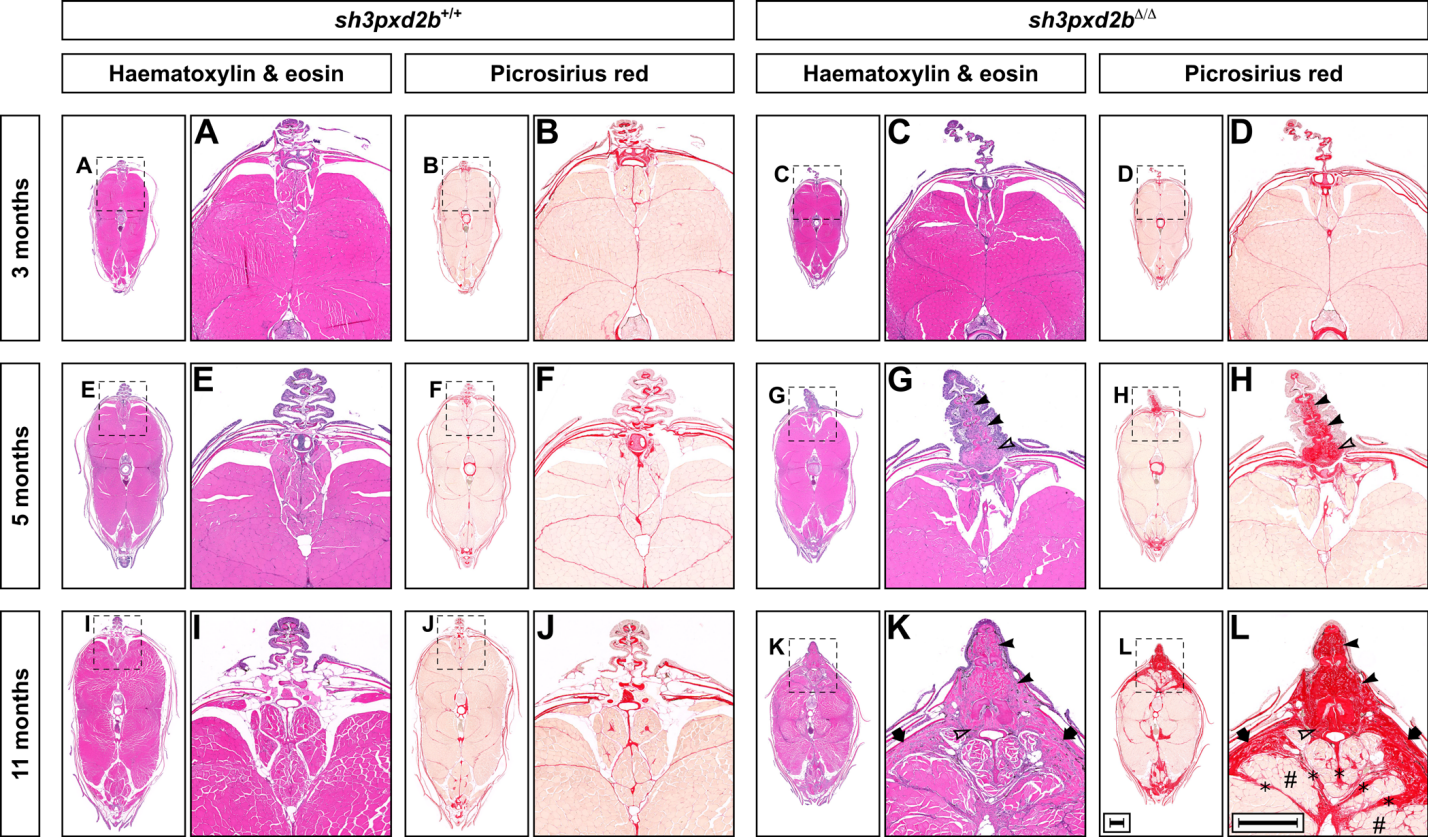
**Adult *sh3pxd2b*^Δ/Δ^ zebrafish develop cutaneous fibrosis that worsens over time.** Representative H&E and PSR stained transverse sections at the level of the dorsal fin of *sh3pxd2b*^Δ/Δ^ mutant fish and *sh3pxd2b*^+/+^ WT clutch mates at the ages of 3, 5 and 11 months, selected from five assessed fish per genotype per time point, imaged with a Zeiss AxioImager Z.2 slide scanner. At 3 months of age, transverse sections of mutants (C,D) and their WT clutch mates (A,B) are indistinguishable. At the age of 5 months, mutants have accumulated an increased amount of connective tissue at the base of the dorsal fin between the lepidotrichiae (G, solid arrow heads) and around the distal radial (outlined arrow head) as compared to their WT clutch mates (E), primarily consisting of collagen (F,H). At 11 months of age, the base of the dorsal fin in mutants is wider than their WT clutch mates and completely filled with collagen (I–L). In addition, collagen accumulates in the stratum compactum (arrows), myosepta (asterisks), and between individual muscle fibres (hash signs) in mutants. Scale bars equal 500 µm.

Given the similarities in cranial abnormalities between the novel *pretzel* model and our previously reported *mmp14a/b* KO zebrafish, we next set out to assess the *pretzel* skeletal phenotype in detail. Similar to our previous studies, we subjected five individuals per genotype per time point to microcomputed tomography (µCT) scanning (De Vos et al., 2018). These radiographic studies clearly demonstrated kyphoscoliosis that develops after the age of 5 months in *sh3pxd2b*^Δ/Δ^ mutants (Fig. 2A, arrow; Fig. S2). Contrary to our expectations (based on the *mmp14a/b* KO fish), *sh3pxd2b*^Δ/Δ^ fish have an increased overall tissue density compared to their WT clutch mates, which is significant at 11 months of age (1234.2 mg cm^−3^ versus 1185.5 mg cm^−3^, *P*=0.0077; Fig. 2Bii), but not before (1186.8 mg cm^−3^ versus 1169.3 mg cm^−3^; *P*=0.1625). In particular, the skin of 11-month-old mutants was denser, appearing as a meshwork on the µCT 3D render (Fig. 2A, arrow heads). As such, we assessed the histology of *pretzel's* skin and dorsal fin. At 3 months of age, transverse sections of *sh3pxd2b*^Δ/Δ^ fish at the level of the dorsal fin were indistinguishable from those of WT clutch mates (Fig. 3A–D), in concordance with Fig. 1. At the age of 5 months, haematoxylin and eosin (H&E) stained transverse sections demonstrated accumulation of connective tissue at the base of the dorsal fin in mutants (Fig. 3G), around the distal radial (outlined arrow head) and the lepidotrichiae (fin rays, solid arrow heads). Picrosirius red (PSR) staining revealed that this connective tissue mainly consists of collagen (Fig. 3H). This collagen accumulation was absent in WT clutch mates (Fig. 3E,F). At 11 months of age, collagen accumulation in *sh3pxd2b*^Δ/Δ^ fish increased and expanded into the base of the dorsal fin (Fig. 3K,L). In addition, collagen accumulation was present in the dermal stratum compactum (Fig. 3L, arrows), myosepta (asterisks), and in between individual muscle fibres (hash signs). Midsagittal sectioning of the 11-month-old mutants shown in Fig. 3 also demonstrated the presence of extensive collagen accumulation near the dorsal and anal fins (Fig. S3A), but no overt bone abnormalities as seen in the *mmp14a/b* KO fish (De Vos et al., 2018). More lateral sagittal sections demonstrated that the inner ear of *sh3pxd2b*^Δ/Δ^ fish is indistinguishable of that of WT clutch mates (Fig. S3B). We did not observe any overt cardiac abnormalities in the mutant fish. As *sh3pxd2b*^Δ/Δ^ fish did not have an overt eye phenotype as observed in multiple individuals with FTHS, we did not perform additional histological analysis of the eye (Bendon et al., 2012; Chang et al., 2017; Hamel et al., 1995; Iqbal et al., 2010; Maas et al., 2004; Mégarbané et al., 1997; Ratukondla et al., 2020; Ter Haar et al., 1982; Wilson et al., 2014; Zrhidri et al., 2017). The fin and flank fibrosis in *sh3pxd2b*^Δ/Δ^ fish has functional consequences. As shown in Movie 1, 1-year-old mutants have a more rigid swimming pattern, keeping their dorsal and pectoral fins in a more or less fixed position, and turning primarily by tail movement.

### (Sub)cutaneous fibrosis of the *pretzel* model cannot be induced by cutaneous wounding

The *sh3pxd2b*^Δ/Δ^ fish spontaneously developed cutaneous and musculoskeletal fibrosis over the course of many months, yet it was unclear when and by what the fibrotic changes were triggered. Given the location where fibrosis initially became apparent, i.e. the base of the dorsal fin, we considered that repeated microtrauma might be responsible. As such, we hypothesised that in the *pretzel* fish, macrotrauma might trigger fibrosis as well. To test this hypothesis, we assessed whether *pretzel* fish responded to cutaneous wounding by excessive collagen deposition, i.e. scarring. We inflicted full-thickness flank wounds in 5-month-old *sh3pxd2b*^Δ/Δ^ fish and WT clutch mates (Fig. S4Ai,ii and S4Bi,ii), and assessed wound closure and collagen deposition 4 and 10 days post wounding as previously described by Richardson and colleagues (2013). After 4 days, wounds in both mutants and WT fish were closed, and contained melanophores (Fig. S4Aiii,iv) that had migrated out of the neighbouring stripes. In addition, there was a strong increase in (sub)cutaneous collagen deposition in both genotypes (Fig. S4Av,vi). After 10 days, both melanophores and iridophores could be observed in the wound (Fig. S4Biii,iv). However, the (sub)cutaneous collagen deposition underlying the wounded area was comparable between genotypes (Fig. S4Bv,vi) and similar to the surrounding unwounded area. Note that the average wound area did not significantly differ between the four subgroups (Fig. S4C). In conclusion, this cutaneous wounding assay demonstrated that collagen remodelling, following acute macrotrauma, is not impaired in the *pretzel* fish.

## DISCUSSION

Skeletal abnormalities form the most prominent features shared by the disorders within the DECOR spectrum (De Vos et al., 2019). Similar to murine and zebrafish models previously reported by us and others, our novel *sh3pxd2b*^Δ/Δ^ mutant *pretzel* phenocopies parts of the DECORS skeletal phenotype (Curtain and Donahue, 2007; De Vos et al., 2018, 2019; Du et al., 2013; Dülk et al., 2016; Holmbeck et al., 1999; Iqbal et al., 2010; Mao et al., 2009; Vas et al., 2019; Zhou et al., 2000). Adult *pretzel* fish are slightly smaller than their WT siblings, have a shorter, up-tilted skull, and develop kyphoscoliosis that worsens over time, likely due to increasing musculoskeletal fibrosis. Until young adult age, the *pretzel* phenotype looks like a mild variant of the *mmp14a/b* KO fish, in which the skeletal abnormalities including stunted growth, cranial up-tilt, and aberrant skull shape are more pronounced (De Vos et al., 2018). However, the cartilage and bone abnormalities observed in *mmp14a/b* KO fish were absent in *pretzel* crispants (De Vos et al., 2018).

Apart from skeletal abnormalities, multiple DECORS patients with *SH3PXD2B* mutation or deletion have been reported to develop fibrotic changes in soft tissues, including generalised dermal fibrosis, subcutaneous nodules, and joint contractures (Bendon et al., 2012; Borrone et al., 1993; Chang et al., 2017; Iqbal et al., 2010; Mégarbané et al., 1997; Ratukondla et al., 2020; Wilson et al., 2014; Zrhidri et al., 2017). Together with skeletal abnormalities, contractures frequently result in debilitating malformations. In contrast to the relatively mild skeletal abnormalities, our *pretzel* fish primarily phenocopies the fibrotic changes of DECORS. The *pretzel* fish develops dermal and musculoskeletal fibrosis from 5 months of age onwards, which gradually worsens. This fibrosis is initially present around the insertion of the dorsal, anal and pectoral fins, and gradually expands from these locations. Subsequent contraction seems to underlie the grotesque malformations observed in the *pretzel* fish, including kyphoscoliosis, abdominal constriction and lateral folding. As in DECORS patients, the fibrosis and contractures impair the fish's mobility (Bendon et al., 2012; Borrone et al., 1993; Chang et al., 2017; Iqbal et al., 2010; Mégarbané et al., 1997; Ratukondla et al., 2020; Wilson et al., 2014; Zrhidri et al., 2017). Histologically, the observed fibrosis in *pretzel* fish resembles that observed in DECORS patients. The *pretzel* fish develop generalised fibrotic thickening of the stratum compactum, similar to diffuse dermal fibrosis seen in humans, and deeper fibrosis in between individual myocytes and adipocytes, the latter as observed in fibrocollagenous nodules of DECORS patients with *SH3PXD2B* or *MMP2* mutation, respectively (Al Aqeel et al., 2000; Borrone et al., 1993). Of note, we previously noticed stiff pectoral fins in a subset of adult *mmp14a/b* KO fish (De Vos, 2018). However, histological analysis did not reveal overt collagen deposition in or around the pectoral fins; instead, an aberrant scapula shape and subscapular muscle hypertrophy was thought to be responsible for the observed pectoral stiffness (De Vos, 2018).

As the *pretzel* fish develops dermal fibrosis at adult age over a period of months, we wondered whether the fibrosis could be due to microtrauma causing abnormal collagen deposition. Hence, we hypothesised that macrotrauma, for instance in the form of full-thickness cutaneous wounding, would result in (dermal) fibrosis. As reviewed by Richardson and colleagues, similar processes are involved in healing of cutaneous wounds in zebrafish and humans (Richardson et al., 2013; Richardson, 2018). Both humans and zebrafish form granulation tissue at the wound site, which is characterised by increased deposition of collagen (Richardson et al., 2013; Richardson, 2018). As in humans, this collagen accumulation is temporary in zebrafish, peaking at 4 days post wounding (dpw) and regressing to pre-wounding levels in the subsequent week (Richardson et al., 2013). Moreover, scar formation is minimal in zebrafish (Richardson et al., 2013). Given the role of *sh3pxd2b* in ECM remodelling and the spontaneous fibrosis in *pretzel* fish, we reasoned that the *pretzel* fish might scar upon cutaneous wounding. However, the vast majority of granulation tissue was removed 10 dpw in both *pretzel* fish and their WT siblings, indicating that macrotrauma does not result in abnormal collagen deposition in *pretzel* fish.

Taken together, our novel *pretzel* zebrafish model for *SH3PXD2B* related DECORS stresses the central role for SH3PXD2B in collagen homeostasis. Although the exact mechanism by which *pretzel* fish form fibrosis remains to be elucidated, our results suggest that it differs from scarring following cutaneous wound healing, as the latter is not affected in the *pretzel* fish. Unlike humans, where DECORS patients with mutations in different genes cannot be distinguished clinically, our zebrafish models with truncating mutations in either *mmp14a/b* or *tks4* show phenotypic differences (De Vos, 2018; De Vos et al., 2018). Whereas *mmp14a/b* KO primarily affects bone, *sh3pxd2b* mutation primarily affects connective tissue. This discrepancy in phenotypes suggests that in humans epistasis is more complete than in zebrafish. In addition, this discrepancy suggests that in fish, as opposed to humans, there are differences in spatiotemporal dependence of ECM remodelling on either *sh3pxd2b* or *mmp14a/b*. This apparent difference in ECM remodelling is striking, as Mmp14a/b are thought of as downstream effectors of Sh3pxd2b in ECM remodelling at the podosome membrane (Buschman et al., 2009). As such, one possible explanation for the observed discrepancy between mutant phenotypes could be differences in the dependence on podosomes for ECM remodelling in different tissues between species. The *pretzel* model presented here can be used to further delineate the underlying mechanism of the fibrosis observed in DECORS, as well as screening and subsequent development of novel drugs targeting DECORS-related fibrosis.

## MATERIALS AND METHODS

### *In silico* analyses

*Homo sapiens SH3PXD2B* (NM_001017995) and *Danio rerio sh3pxd2b* (XM_005170288.3) genomic DNA (gDNA), exon, and protein sequences were accessed through the UCSC Genome Browser [University of California, Santa Cruz, CA, USA; human assembly December 2013 (GRCh38/hg38) and zebrafish assembly May 2017 (GRCz11/danRer11); URL: genome.ucsc.edu/cgi-bin/hgGateway; last accessed January 2020]. Human SH3PXD2B protein domains were retrieved from the Universal Protein Database (UniProt; URL: www.uniprot.org; last accessed May 2018; accession number A1X283). Zebrafish Sh3pxd2b protein domains were mapped by aligning the human SH3PXD2B protein sequence and the predicted zebrafish Sh3pxd2b protein sequence with PRALINE (Centre for Integrative Bioinformatics VU, Vrije Universiteit Amsterdam, Amsterdam, the Netherlands; URL: www.ibi.vu.nl/programs/pralinewww; last accessed January 2020).

### Zebrafish husbandry

*Danio rerio*, AB strain, were housed in the zebrafish facilities of the A*STAR Institute of Molecular and Cell Biology and of the Nanyang Technological University as previously described (De Vos et al., 2018). In brief, embryos and larvae were grown in E3 medium at 28.5°C in the dark until 5 dpf, after which they were kept in a fixed light/dark (14 h/10 h) circadian rhythm and feeding schedule. At 14 dpf, larvae were transferred to a closed water system. From 28 dpf onwards, fish were kept at 27.5°C. Mutant zebrafish were generated under IACUC licence 140924, bred and maintained under IACUC licence 161172, and wounded under IACUC licence A18010, in accordance with National Advisory Committee for Laboratory Animal Research (NACLAR) guidelines.

### Generation of gRNA and Cas9 RNA for CRISPR/Cas9 genome editing

Optimal CRISPR target sites in zebrafish *sh3pxd2b* (XM_003200907.4), compatible with T7 transcription, were determined by use of ZiFiT Targeter (Version 4.2; Zinc Finger Consortium; URL: zifit.partners.org/ZiFiT) and blasted against the *D. rerio* genome to screen for possible non-specific targets by CRISPRscan (Giraldez Lab, Yale University; URL: www.crisprscan.org, last accessed both websites in May 2014) (Moreno-Mateos et al., 2015; Sander et al., 2007, 2010). A target in exon 13 of *sh3pxd2b* that was predicted not to result in non-specific targeting, 5′-TAGCTGACTTCCAGACAACC-3′ (reverse orientation), was ordered as part of a gBlocks^®^ Gene Fragment (Integrated DNA Technologies, Coralville, IA, USA). This fragment contained a 5′-CATTATGGTGAAAGTTGGAAC-3′ forward (Fw) and 5′-AAAAGCACCGACTCGGTGCCAC-3′ reverse (Rv) primer sequence at its 5′ and 3′ end, respectively, in addition to a T7 promoter immediately 5′ adjacent to the target site sequence. Guide RNA (gRNA) was generated from the Gene Fragment as described before (De Vos et al., 2018). Specifically, gBlocks DNA was amplified using PrimeSTAR^®^ Max DNA Polymerase (TaKaRa Bio Inc., Kyoto, Japan, R045B) in DNA Engine Dyad^®^ (Bio-Rad, Hercules, CA, USA, PTC-0220) with cycling parameters: initial denaturation (98°C, 60 s), followed by 25 cycles of denaturation (98°C, 10 s), annealing (55°C, 5 s), and extension (72°C, 5 s). Polymerase chain reaction (PCR) product was checked on a 1% (*w/v*) agarose (1st BASE, Singapore, Singapore, BIO-1000-500 g) in 1X tris-acetate-ethylenediaminetetraacetic acid (EDTA) buffer (TAE, 1st BASE; BUF-3000-50X4L) gel, visualised by SYBR™ Safe DNA Gel Stain (Thermo Fischer Scientific Inc., Waltham, MA, USA, S33102) on a ChemiDoc™ XRS+ System (Bio-Rad, 1708265) operated by Image Lab™ Software (5.1 build 8, Bio-Rad). The amplicon was purified with the Wizard^®^ SV Gel & PCR Clean-Up System (Promega, A9282), and transcribed *in vitro* into gRNA by MEGAshortscript T7 kit (Thermo Fisher Scientific, AM1354) as per manufacturer's protocol. RNA was checked by running it on a 2% (*w/v*) agarose (1st Base) in 1X TAE (1st Base) gel. The pCS2-nCas9n vector (a gift from Dr T.J. Carney) was linearised by *NotI* and transcribed into *Cas9* RNA *in vitro* by mMESSAGE mMACHINE™ SP6 Transcription Kit (Thermo Fisher Scientific, AM1340) as per manufacturer's protocol. RNA was checked on a 0.8% (*w/v*) agarose (1st Base) in 1X TAE (1st Base) gel.

### CRISPR/Cas9 genome editing of *sh3pxd2b* in zebrafish

The yolk cell of *D. rerio* AB WT zygotes was injected with 0.750 ng gRNA and 0.375 ng *Cas9* RNA in a total volume of 2.5 nl of 1X Danieau's solution [58 mM NaCl (Promega, H5273), 0.7 mM KCl (Merck, 101985), 0.4 mM MgSO_4_ (Sigma-Aldrich, M2643), 0.6 mM Ca(NO_3_)_2_ (Sigma-Aldrich, C1396), 5.0 mM HEPES, pH 7.6 (Sigma-Aldrich, 94717)] / 1X PhenolRed (Sigma-Aldrich, P0290) in dH_2_O using glass needles and an IM-300 Microinjector (Narishige, Tokyo, Japan) (Rosen et al., 2009). To test for successful targeting, a proportion of the injected embryos was lysed at 24 hpf at 55°C for 5 h in 25 ml of lysis buffer [10 mM Tris (1st BASE, BIO-1400-500 ***g***), 50 mM KCl (Merck, 102525M), 0.3% (*v/v*) Tween^®^ 20 (Promega, H5151), 0.3% (*v/v*) Nonidet P40 substitute (United States Biological, Salem, MA, USA; N3500) and 0.488 mg/ml proteinase K (Thermo Fisher Scientific, catalogue number EO0491) in dH_2_O (pH set to 8.3)]. After heat-inactivation of proteinase K at 95°C for 10 min, the lysate was diluted 1:1 with dH_2_O and a 437 bp region around the *sh3pxd2b* target site in the gDNA was amplified by short genomic PCR with Taq DNA polymerase (Roche, 11578553001) using Fw (5′-CCTGCTCCATAGGGTAAACCTG-3′) and Rv (5′-CAATGAATGTGACTGGAGCCCAG-3′) primers and 1 µl unpurified lysate as template, with cycling parameters: initial denaturation (94°C, 120 s); 30 cycles of denaturation (94°C, 30 s), annealing (54°C, 30 s), and extension (72°C, 60 s); final extension (72°C, 420 s). The PCR product was diluted 1:10 in dH_2_O and subjected to direct Sanger sequencing using BigDye™ Terminator Ready Reaction Mix (version 3.1, Thermo Fisher Scientific, 4337455) with cycling parameters: initial denaturation (96°C, 180 s); 35 cycles of denaturation (96°C, 30 s), annealing (55°C, 15 s), and extension (60°C, 240 s). Extension products were purified with Agencourt CleanSEQ Sanger Sequencing Dye Terminator Removal (Beckman Coulter, Fullerton, CA, USA, A29161) as per manufacturer's protocol, and chromatography was performed with a 3730XL sequencer (Thermo Fisher Scientific). Chromatography reads were screened for double peaks in SnapGene (version 3.3.4, Clontech Laboratories, Mountain View, CA, USA), mutations checked for frameshift in the Poly Peak Parser webtool (Yost lab, Salt Lake City, UT, USA), and identified frameshift mutations were checked manually (Hill et al., 2014). At 3 months of age, F0 injected fish were intercrossed and a proportion of their F1 offspring was genotyped for *sh3pxd2b* as described above. When heterozygous mutations were detected, F1 clutch mates were grown and fin-clip genotyped at the age of 2 months. F1 *sh3pxd2b*^Δ*/+*^ heterozygotes were intercrossed to produce F2 *sh3pxd2b*^Δ/Δ^ homozygote mutant fish.

### Generation of zebrafish cDNA

Five *sh3pxd2b*^+/+^ and five *sh3pxd2b*^Δ/Δ^ 8-week-old juvenile zebrafish of average size were euthanised by overdose of ethyl 3-aminobenzoate methanesulfonate (Tricaine, Sigma-Aldrich, A5040) in NaHCO_3_-buffered system water buffered (pH 7.0-7.5; Sigma-Aldrich, S5761), cut into small pieces into one individual tube per fish, snap frozen on dry ice and homogenised with a pestle and subsequently a 23G needle (Becton, Dickinson and Company, Franklin Lakes, NJ, USA; 305143) in a total volume of 3 ml TRIzol Reagent (Thermo Fisher Scientific, 15596026) per fish. After adding 600 µl chloroform (Kanto; 07278-00), samples were vortexed for 15 s and incubated for 180 s at room temperature (RT). Samples were centrifuged at 11,000 rpm for 5 min at 4°C, after which the aqueous phase was incubated with 3 ml isopropanol (Merck, 1.09634.2500) for 10 min at RT in a fresh tube. Samples were centrifuged at 10,000 rpm for 15 min at 4°C, after which the supernatant was discarded and the pellet rinsed in 3 ml ice-cold 75% (*v/v*) ethanol in diethyl pyrocarbonate (DEPC)-treated water (Sigma-Aldrich, 159220-25G). Samples were centrifuged at 7500 rpm for 5 min at 4°C, the supernatant removed, and the air-dried pellet dissolved in 100 µl RNAse-free water (Qiagen, Hilden, Germany, 129112). DNA was digested by RNase free DNase Kit (Qiagen, 79254). The samples were column purified by Qiagen RNeasy Mini Kit (Qiagen, 74104) as per manufacturer's protocol, after which whole zebrafish RNA was eluted in 20 µl of RNAse-free water. RNA concentration was assessed by NanoDrop™ One Spectrophotometer (Thermo Fisher Scientific, ND-ONE-W), and RNA quality was confirmed by running it on a 1% (*w/v*) agarose (1st Base) in 1X TAE (1st Base) gel. Complementary DNA was synthesised by high-capacity cDNA reverse transcription kit (Thermo Fisher Scientific, 4368813) as per manufacturer's protocol, with cycling parameters: 25°C for 10 min, 37°C for 120 min, and 85°C for 5 min.

### Analysis of the CRISPR-induced *sh3pxd2b* mutation on mRNA expression

Primers for quantitative PCR (qPCR) were tested at various concentrations (1.95-500 nM) on 2-month-old *sh3pxd2b*^+/+^ juvenile cDNA in technical triplicate. Quantitative PCR was performed using SYBR Select Master Mix (Thermo Fisher Scientific, 4472919) and a StepOne™ Real-Time PCR System (Thermo Fisher Scientific, 4376357) as per manufacturer's protocol and cycling parameters: initial denaturation (95°C, 120 s); 40 cycles of denaturation (95°C, 3 s), annealing (58°C, 10 s), extension (72°C, 30 s), followed by data collection. After the qPCR reaction, triplicates of lowest primer dilutions were pooled and column-purified by MinElute PCR Purification Kit (Qiagen, 28006). To ensure only a single reaction product was generated, the reaction product was run on a 3% (*w/v*) agarose (1st Base) in 1X TAE (1st Base) gel and furthermore assessed by direct Sanger sequencing (AITBiotech Pte Ltd, Singapore) with both the respective Fw and Rv primer. Primer pairs selected for analysis of *sh3pxd2b* expression levels were 5-CCTTCAGATGGAGCTTCTGG-3′ (Fw) and 5- TTTGGTGGGTTCAGGTCTTC-3 (Rv). Expression levels of mutant *sh3pxd2b* mRNA were normalised to those of *β-actin* (with 5′-CGAGCAGGAGATGGGAACC-3′ (Fw) and 5′-CAACGGAAACGCTCATTGC-3′ (Rv) primers), and denoted relative to the expression level of WT *sh3pxd2b* following ΔΔCt method (De Vos et al., 2018).

### Cell culture

MRC-5V1 immortalised human foetal lung fibroblasts (University of Sussex, Brighton, UK) were cultured in 2D as described before (De Vos et al., 2018). In brief, cells were grown in log phase at 37°C in 100% humidity and 5% CO_2_ in high glucose Dulbecco's Modified Eagle Medium (DMEM, GE Healthcare Life Sciences, Pittsburgh, PA, USA, SH3024.01) that contained 10% (*v/v*) Fetal Bovine Serum (FBS; GE Healthcare Life Sciences, Pittsburgh, Pennsylvania, A15-101), 100 U/ml penicillin and 100 µg/ml streptomycin (Thermo Fisher Scientific, 15140122). Cell type was verified by morphology and vimentin expression upon their acquisition in our lab. Cells were tested for mycoplasma contamination prior to cryopreservation. A vial was thawed and cultured in log phase for at least six passages prior to their use in experiments.

### Cloning of pCS2+ EGFP-sh3pxd2b-HA plasmids

To analyse the effects of the CRISPR/Cas9-induced *sh3pxd2b* frameshift mutation on the protein level, expression vectors encoding the C-terminus of either WT or mutant Sh3pxd2b with an amino (N-) terminal enhanced green fluorescent protein (EGFP) and carboxyl (C) terminal Haemagglutinin (HA) tag were generated. WT and mutant *sh3pxd2b* cDNA, respectively, was obtained from 21-day-old embryos, pooled per genotype, as described above. Exon 13 (excluding the first 15 base pairs at 5′) and exon 14 of either WT or mutant *sh3pxd2b* were amplified from WT or mutant cDNA, respectively, by PCR with High Fidelity Phusion Flash (Thermo Fisher Scientific, F548S) and primers 5′-CGAGGAGTAAACCTACCTAAACC-3′ (Fw) and 5′-AGGTTTCTTGGTCAGGTAATTG-3′ (Rv) with parameters: initial denaturation (95°C, 120 s); 30 cycles of denaturation (94°C, 30 s), annealing (58°C, 30 s), and extension (72°C, 60 s); final extension (75°C, 600 s). To check the result of the PCR reaction, 5 µl of reaction product was ran on a 1% (*w/v*) agarose (1st Base) in 1X TAE (1st Base) gel, after which the remaining reaction product was run on a separate gel for extraction of the band of correct size. After cutting out the band, DNA was extracted by QIAquick PCR Purification Kit (Qiagen, 28106) and eluted in nuclease-free water by MinElute PCR Purification Kit (Thermo Fisher Scientific). The WT and mutant PCR product were cloned into the pTOPO vector as per zero blunt TOPO PCR cloning kit for sequencing (Thermo Fisher Scientific, K2895-20), and the insert verified by sequencing. The WT and mutant *sh3pxd2b* sequences were subsequently amplified from the respective pTOPO vectors, with the addition of a 3′ HA-encoding sequence by use of the primers 5′-GAATTCCTACCTAAACCTCCAGTGCCCCC-3 (Fw) and 5′-CTCGAGTCAAGCGTAATCTGGAACATCGTATGGGTAAGGTTTCTTGGTCAGGTAATTGG-3′ (Rv, with HA-encoding sequence underlined). An EGFP-encoding sequence was amplified from the pCS2+ N-EGFP-mVangl2 vector with primers 5′-GGATCCGCCACCATGGTGAGCAAGGGC-3′ (Fw) and 5′-GAATTCACCACCACCACCACCACCCTTGTACAGCTCGTCCATGCCG-3′ (Rv), and both the *sh3pxd2b-HA* and *EGFP* coding sequences were cloned into the pCS2+ backbone with the Quick Ligation Kit (NEB, M2200S). To verify correct insertion into the pCS2+ backbone, a diagnostic restriction digestion with BamHI (New England Biolabs, Ipswich, MA, USA, R0136S) was performed, and the digestion products checked for size on a 1% agarose (1st Base) in 1X TAE (1st Base) gel. Subsequently, the sequence of the insert was verified by direct Sanger sequencing.

### Analysis of the CRISPR-induced *sh3pxd2b* mutation on the protein level

Twenty-four hours after seeding 100,000 MRC-5V1 cells/ml DMEM in a six-well plate (Corning; CLS3516), cells were transfected with 2 µg of either WT or mutant pCS2+ EGFP-Sh3pxd2b-HA vector, or pQCXIB 3HA-EGFP vector (described previously) as positive control, and 6 µl jetPRIME^®^ (Polyplus-transfection^®^ SA, Illkirch-Graffenstaden, France; 144-15) per well as per manufacturer's protocol (De Vos et al., 2018). Twenty-four hours post-transfection, transfection efficiency was assessed by estimating the percentage of green fluorescent cells by use of an EVOS™ FL Auto 2 Imaging system (Thermo; AMAFD2000). Trypsinised cells of six wells were pooled and pelleted at 300×g for 5 min and rinsed in PBS 1X, after which pelleted cells were lysed on ice for 30 min in 100 µl of lysis buffer composed of 1% (*v/v*) Nonidet P40 Substitute (Sigma-Aldrich), 150 mM NaCl (Promega), 250 mM Tris pH 7.5 (1st BASE; BUF-1415-500ml-pH7.5), PhosSTOP™ Phosphatase Inhibitor Cocktail (Roche, 04906837001), cOmplete™ Mini EDTA-free Protease Inhibitor Cocktail (Roche; 11836170001), 5 mM CaCl_2_ (Kanto) and 1 µl Micrococcal Nuclease (NEB; M0247S). Whole cell lysate was centrifuged at top speed for 30 min at 4°C, after which the protein concentration of the supernatant was assessed with the Pierce™ BCA Protein Assay (Thermo Fisher Scientific, 23227) and a Varioskan™ LUX microplate reader (Thermo Fisher Scientific) operated by SkanIt™ RE software (Thermo Fisher Scientific, version 6.0.1). Samples (50 µg each) were mixed with an appropriate volume of 5X SDS Page loading dye (iNtRON Biotechnology, Seongnam, Korea; IBS-BS002), heated to 99°C for 5 min, and ran next to 5 µl of Chameleon Duo Pre-stained Protein Ladder (LI-COR Biosciences, Lincoln, NA, USA, 978-16526) on 10% Mini-PROTEAN^®^ TGX™ gels (Bio-Rad; 456-1034) in 0.1 M Tris-base (Formedium, Hunstanton, UK; TRIS01) / 0.1 M glycine (Melford, Ipswich, UK; G0709) / 0.025% (*w/v*) sodium dodecyl sulphate (SDS; Bio-Rad; 161-0418) in dH_2_O. Separated proteins were transferred to Immobilon^®^-FL PVDF membranes (Merck; IPFL10100) overnight at 30 mA at 4°C in 25 mM Tris / 192 mM glycine / 20% (*v/v*) methanol (VWR, Radnor, PA, USA, 20847.307) / 0.02% (*w/v*) SDS in dH_2_O. Even loading of samples was assessed by Revert™ Total Protein Stain (LI-COR, 926-11010) as per kit and imaged with an Odyssey^®^ CLx Imaging System (LI-COR), controlled by Image Studio™ (LI-COR, version 5.2). The membranes were blocked in Intercept^TM^ blocking buffer PBS (LI-COR, 927-70001) for 1 h at RT with gentle agitation, and incubated with rabbit anti-HA (Cell Signaling Technology, Danvers, MA, USA, 3724, clone C29F4, lot 10/2019, used at 1:1000 dilution) and mouse anti-GFP (Roche Applied Science, Penzberg, Germany, 11814460001, clones 7.1 and 13.1, lot 10126200; used at 1:2000 dilution) primary antibodies in Intercept blocking buffer containing 0.1% (*v/v*) Tween 20 overnight at 4°C with gentle agitation (De Vos et al., 2018). Membranes were rinsed in 0.1% (*v/v*) Tween 20 in 1X PBS and incubated with donkey anti-mouse IRDye^®^ 800CW (LI-COR; 925-32212, lot C90108-21, used at 1:15,000 dilution) and goat anti-rabbit IRDye^®^ 680LT (LI-COR; 925-68021, lot C90205-11, used at 1:20,000 dilution) secondary antibodies in Intercept blocking buffer containing 0.1% (*v/v*) Tween 20 and 0.01% (*w/v*) SDS for 1 h at RT, protected from light, after which blots were imaged with the Odyssey CLx imaging system. Brightness and contrast was adjusted equally per channel in Image Studio™ (LI-COR; version 5.2.5).

### Gross anatomy of adult zebrafish

Fish to be imaged were sedated in 200 µg/ml Tricaine in system water. After movement stopped and fish were no longer responsive to touch, yet opercular movement and cardiac beating were still observed, fish were imaged on moist filter paper in a 10 cm petri dish lid with a stereomicroscope. Per genotype, fish were randomly selected, without selecting for sex. For initial screening of F1 and F2 generations for a CRISPR-induced phenotype, fish were imaged with an MZ16 FA stereomicroscope (Leica, Wetzlar, Germany) equipped with a DFC 300 FX Digital Colour Camera (Leica) with 1.4 Mpixel resolution, and fish used for micro-computed tomography (µCT) were imaged with an M80 stereomicroscope (Leica) with Plan 1.0X lens (Leica) and DFC425 Digital Colour Camera (Leica) with 5 Mpixel resolution, both operated with Leica Application Suite software (version 4.9; Leica). Fish used for histological analysis and wounding were imaged with an SMZ25 stereomicroscope (Nikon Corporation, Tokyo, Japan) with P2-MFU Motorized Focus Unit, P2-SHR Plan Apo 0.5X lens with 0.078 NA (Nikon), C-FLED2 light source (Nikon) with P2-FIR ring light (Nikon), P-SXY46 Manual X-Y Stage (Nikon), and Ds-Fi3 5.9 Mpixel camera (Nikon), operated by NIS-Elements software (Nikon, version 5.20.00). Gross anatomy images were taken with indirect illumination under an illumination dome (Kawada and Buffington, 2016). For images of the dorsal fin additionally the extended depth of focus plugin was used. Images taken with the SMZ25 were stitched in NIS Elements; images taken with Leica stereomicroscopes were stitched in Fiji software (U. S. National Institutes of Health, Bethesda, MA, USA; ImageJ version 1.51d) with MosaicJ plugin (Thévenaz and Unser, 2007). Brightness and contrast were adjusted equally (for images in Fig. S2) or to similar background (for images in Fig. 1 and Fig. S4) in Keynote (version 8.1; Apple Inc., Cupertino, CA, USA) Standard length (as defined by Parichy et al., 2009) was measured in lateral view in Fiji software (Parichy et al., 2009). Swimming pattern of zebrafish was recorded with an iPhone 8 Plus (built-in lens with 1.8 NA) at 30 frames per second and 2.1 Mpixel resolution. Movies were edited in Photoshop^®^ (Creative Cloud 2020, release 21.1.1; Adobe Systems Incorporated, San José, CA, USA).

### Wounding of adult zebrafish

Ten average-sized five-month-old *sh3pxd2b*^+/+^ and *sh3pxd2b*^Δ/Δ^ fish were sedated in 200 µg/ml Tricaine in system water as described above and placed on moist filter paper. Under stereoscopic magnification (Nikon), ∼6-9 scales on the left flank were removed by forceps. A ∼0.5 mm incision was made with a scalpel blade, and from here a 1.5×1.5 mm full-thickness cutaneous wound was cut with Dowell scissors (Moria SA, Antony, France; MC26B). Wounded fish were placed in 1 liter of system water without Tricaine, and observed for 5 min until full recovery from the sedation. Fish were subsequently single housed in 3 L of system water on flow, as described above, and monitored daily for any signs of sickness or infection daily. After either 4 or 10 days, fish were fixed in 4% PFA in 1X phosphate buffered saline (PBS, GE, SH30028.03) in dH_2_O for 5 days at 4°C, and sent to the A*STAR Advanced Molecular Pathology Laboratory (AMPL) for histological analysis (see below).

### Microcomputed tomography

Average sized 5- and 11-month-old *sh3pxd2b*^+/+^ and *sh3pxd2b*^Δ/Δ^ fish were euthanised by Tricaine overdose, fixed in 4% PFA as described above, rinsed in dH_2_O, and dehydrated through graded water into ethanol (50–75–100% (*w/v*)) over the course of 3 days at 4°C, after which pure ethanol was refreshed once and fish stored at 4°C until imaging. Microcomputed tomography (µCT) images were acquired as described before (De Vos et al., 2018). Tissue density was calculated from a Hounsfield unit corrected standard curve. Skeletal 3D renders were generated with AMIRA software (FEI, Mérignac Cedex, France) with constant window settings.

### Histology

Average-sized 3, 5- and 11-month-old *sh3pxd2b*^+/+^ and *sh3pxd2b*^Δ/Δ^ fish were euthanised and fixed as described above, decalcified in Osteosoft (Merck; 101728) for 2–3 days depending on the size of the fish and trimmed (anterior of the dorsal fin for midsagittal sections, or the region between dorsal and anal fin for transverse sections). Samples processed for histological analysis as described before (De Vos et al., 2018). In brief, samples were dehydrated, paraffin infiltrated, sectioned midsagittally or transverse, deparaffinised, rehydrated and stained with haematoxylin and either eosin or Picrosirius red. Coverslipped sections were imaged with a Zeiss automated slide scanner with Plan-Neofluor 20×/0.5 Ph2 lens (Zeiss), SSCOPED TL light source and 1.4 Mpixel CoolCube 1 camera (MetaSystems GmbH, Altlussheim, Germany). Images were viewed with VSViewer software (MetaSystems; V2.1.103). Brightness and contrast were adjusted equally in Keynote (Apple Inc.).

### Statistics

Biological replicates of standard length measurements, qPCR ΔΔCt values, tissue density measurements, and wound area measurements were assessed for normal distribution per subgroup (genotype and age) by Shapiro-Wilk test. A two-sampled, non-pooled, two-tailed Student's *t*-test (Statistics Study version 4.31; Statext LLC, Carlstadt, NJ, USA) was used to test for statistically significant differences between mean values of said measurements between subgroups. Scored phenotypical features were assessed for statistically significant differences in frequency of occurrence between subgroups by Fisher Exact test (online tool by Navendu Vasavada, URL: https://astatsa.com/FisherTest/). A *P*-value below 0.05 was used as cut-off point to reject the null hypothesis for all performed statistical tests. All graphs were generated in Microsoft Excel (for Mac 2011, version 14.7.7). Final figures were created in Inkscape software version 0.92 (available at inkscape.org/).

## Supplementary Material

Supplementary information
