# Supplementary material for: The novel zebrafish model pretzel demonstrates a central role for SH3PXD2B in defective collagen remodelling and fibrosis in Frank-Ter Haar syndrome
Source: Biol Open. 2020 Dec 29;9(12):bio054270. doi: 10.1242/bio.054270 (PMC7790187; doi:10.1242/bio.054270)
Supplement: Supplementary information [file biolopen-9-054270-s1.pdf]

Table S1 – Levels of significance for differences in prevalence of phenotypical features between adult *sh3pxd2b*$^{\Delta/\Delta}$ and *sh3pxd2b*$^{+/+}$ zebrafish at different age.

**Feature**	**Age 3 months** (P-value)	**Age 5 months** (P-value)	**Age 11 months** (P-value)
Cranial up-tilt	< 0.0001	< 0.0001	< 0.0001
Kyphoscoliosis	1	< 0.0001	< 0.0001
Dorsal fin fibrosis	1	0.055	< 0.0001
Abdominal constriction	1	1	< 0.0001
Dorsal hyperpigmentation	1	0.24	< 0.0001

Prevalence of phenotypical features as shown in Table 1, tested for significant differences between the two genotypes by Fisher Exact test.

Biology Open • Supplementary information

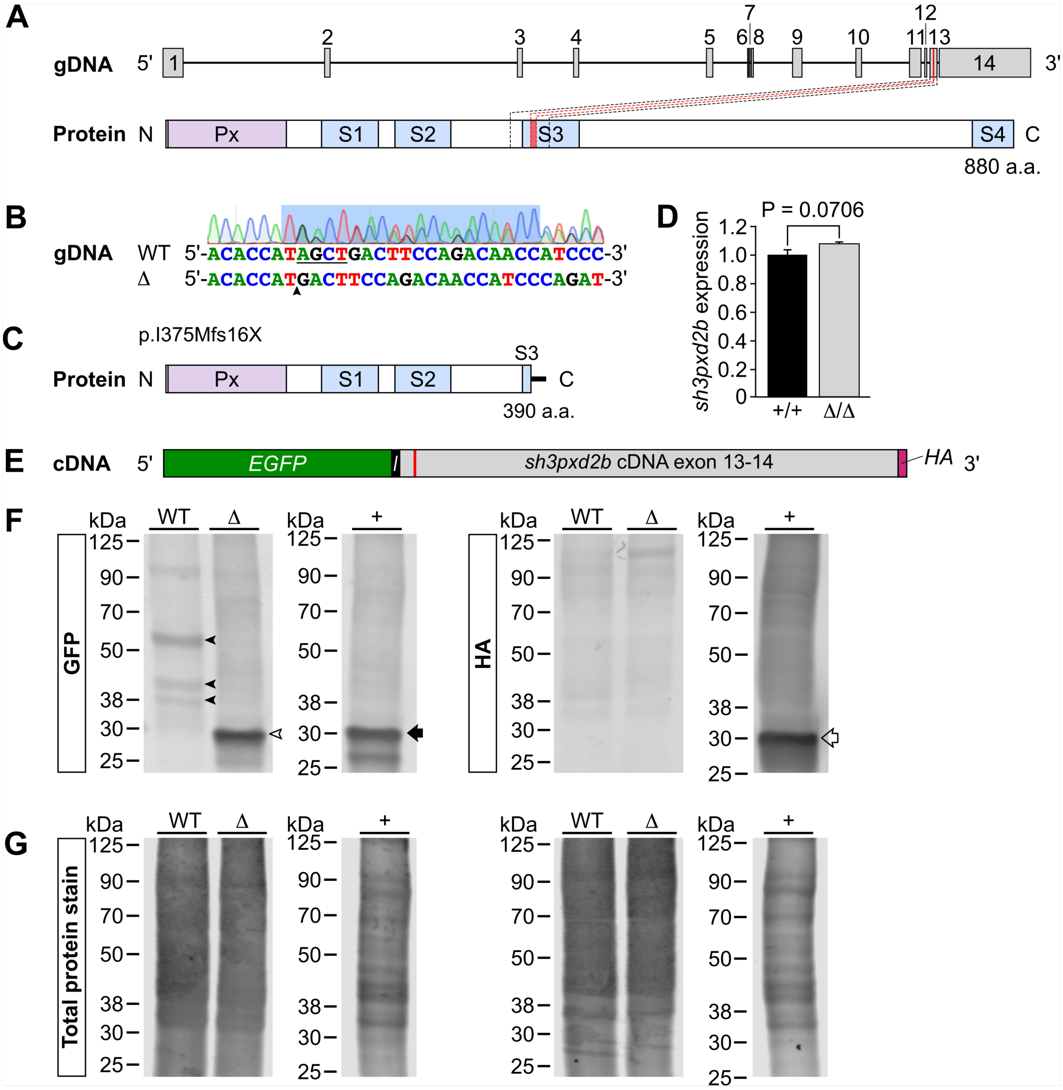

Figure S1 – Editing of *sh3pxd2b* in zebrafish by CRISPR/Cas9. A, *Danio rerio sh3pxd2b* is predicted to encompass 14 exons, together encoding an 880 a.a. protein. Guide RNA was designed to target exon 13 (target site indicated in red, and highlighted in (B) in blue), which encodes the N-terminal part of the third Src Homology 3 domain (S3). **B**, CRISPR/Cas9 editing of zebrafish *sh3pxd2b* resulted in a 4 bp deletion in exon 13 (arrowhead, underlined). **C**, the CRISPR-induced deletion shown in (B) is predicted to result in the truncation p.I375Mfs16X, keeping only the N-terminal 43% of the protein intact. **D**, qPCR analysis of 2-month-old *sh3pxd2b*$^{\Delta/\Delta}$ mutants and *sh3pxd2b*$^{+/+}$ WT clutch mates demonstrated that the mutation shown in (B) had no statistically significant (P = 0.0706) effect on *sh3pxd2b* mRNA expression (relative to *β-actin* expression, normalised to WT expression levels; error bars represent standard error of means of ΔΔCt values of five biological replicates per genotype; P = 0.07 as assessed by two-sampled, non-pooled, two-tailed Student's t-test). **E**, WT or mutant exons 13-14 were cloned into the pCS2+ expression vector, together with a 5' EGFP coding sequence and short linker (l), and a 3' HA coding sequence. Red line indicates the location of the CRISPR-induced mutation in mutant cDNA. **F**, whole cell protein lysate of MRC-5V1 human fibroblasts expressing either the WT or mutant (Δ) construct, or 3HA-EGFP positive control (+), was subjected to immunoblotting with an anti-GFP and anti-HA antibody, species-specific IRDye® secondary antibodies, and visualised with the LI-COR Odyssey® CLx Imaging system (De Vos et al., 2018). EGFP-Sh3pxd2b^{WT} is present as multiple bands between 38-60 kDa (solid arrow heads), while EGFP-Sh3pxd2b$^{\Delta}$ and 3HA-EGFP run around 31 (open arrow head) and 32 kDA (solid and open arrow), respectively. No HA signal can be detected in the Sh3pxd2b fusion protein samples. **G**, Total protein stain indicates equal loading of the Sh3pxd2b fusion protein samples.

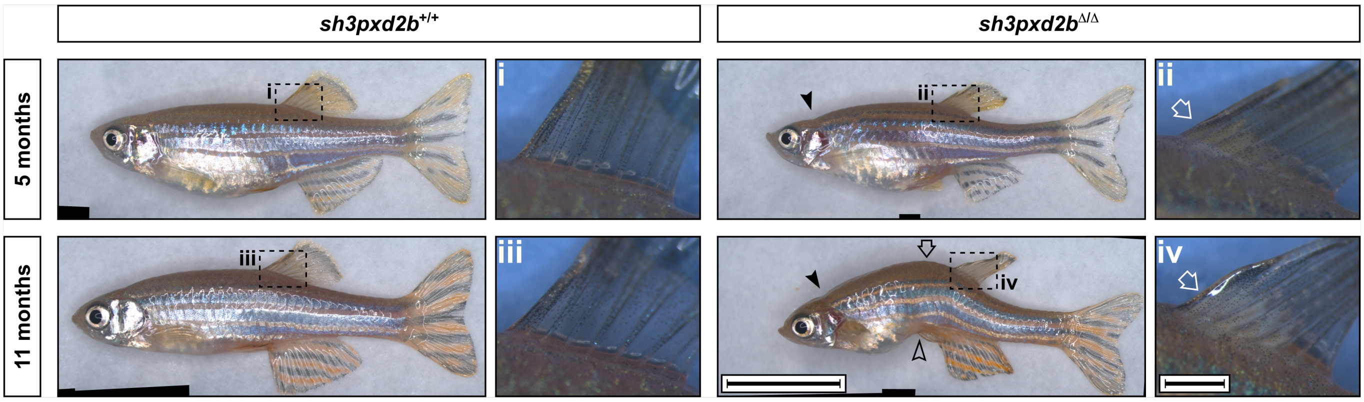

Figure S2 – Gross skeletal phenotype of *sh3pxd2b*$^{\Delta/\Delta}$ zebrafish analysed by µCT. Images were taken with a Leica M80 stereomicroscope. At 5 months of age, mutant fish have an up-tilted head (solid arrow head) and an opaque spot at the base of the dorsal fin (ii, white outlined arrow), which is absent in their WT clutch mates (i). At 11 months of age, mutant fish have a larger opaque spot at the base of the dorsal fin (iv, outlined arrow; compare to i-iii), in addition to kyphosis (outlined arrow) and abdominal constriction (outlined arrow head), as shown in Fig. 1. Scale bar equals 10 mm in overview and 1 mm in dorsal fin close-up.

Biology Open • Supplementary information

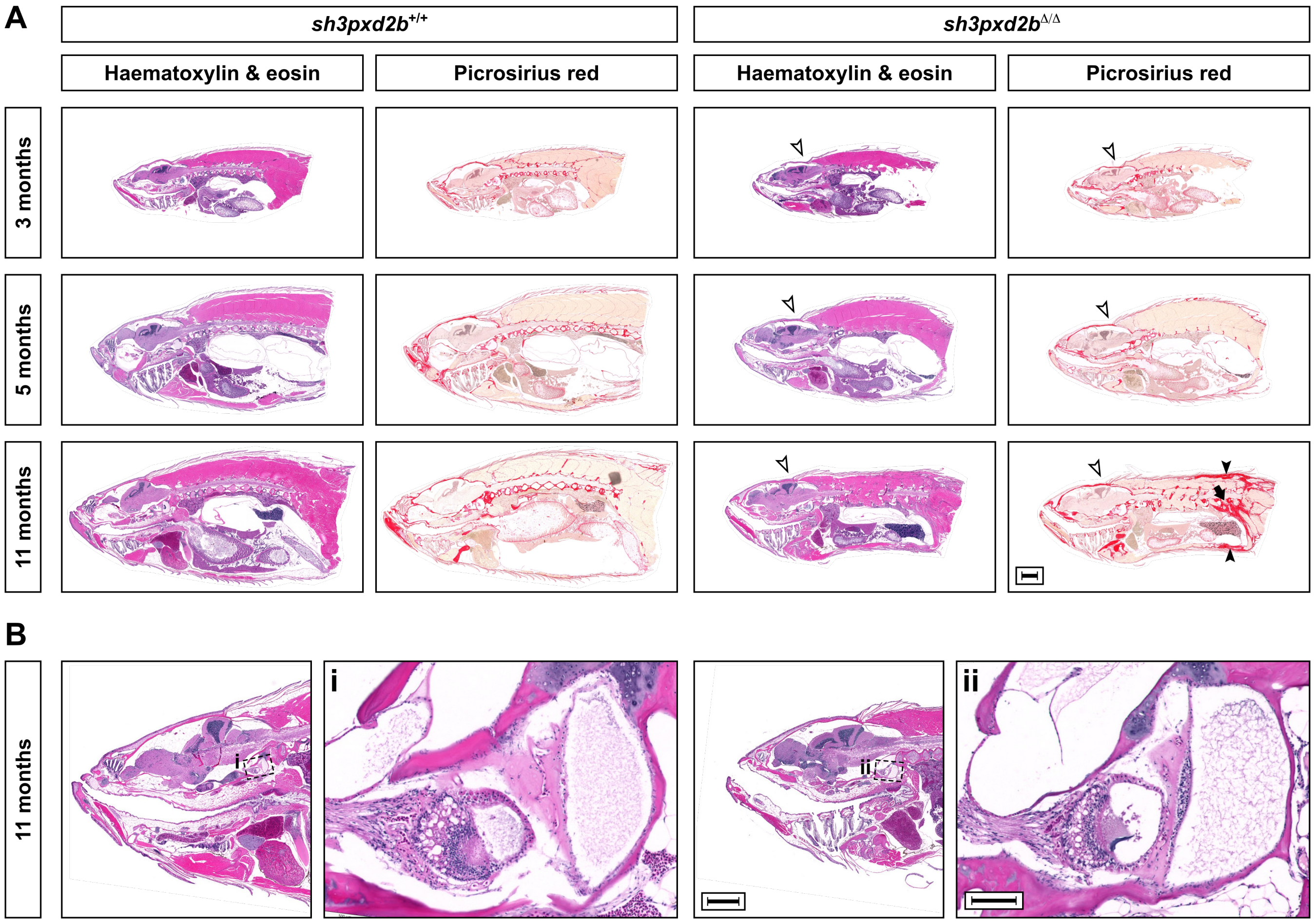

Figure S3 – Adult *sh3pxd2b*$^{\Delta/\Delta}$ zebrafish develop cutaneous fibrosis around the dorsal and anal fin. **A**, H&E and PSR-stained midsagittal sections of the *sh3pxd2b*$^{\Delta/\Delta}$ mutant fish and *sh3pxd2b*$^{+/+}$ WT clutch mates at the age of 3, 5, and 11 months, shown in Fig. 3, imaged with a Zeiss AxioImager Z.2 slide scanner. On midsagittal sections, adult mutants are smaller than their WT clutch mates, and have a smaller, up-tilted head, recognisable by the characteristic dent behind the skull (outlined arrow head). At 11 months of age, an increased collagen content can be observed in the stratum compactum (solid arrow heads) and myosepta (arrow) in the region close to the dorsal fin and the anal fin. **B**, the otolith organ of 11-month-old mutants (ii) does not show overt abnormalities when compared to that of WT clutch mates. Scale bar equals 1 mm in overview and 100 µm in inner ear close-ups i-ii.

Biology Open • Supplementary information

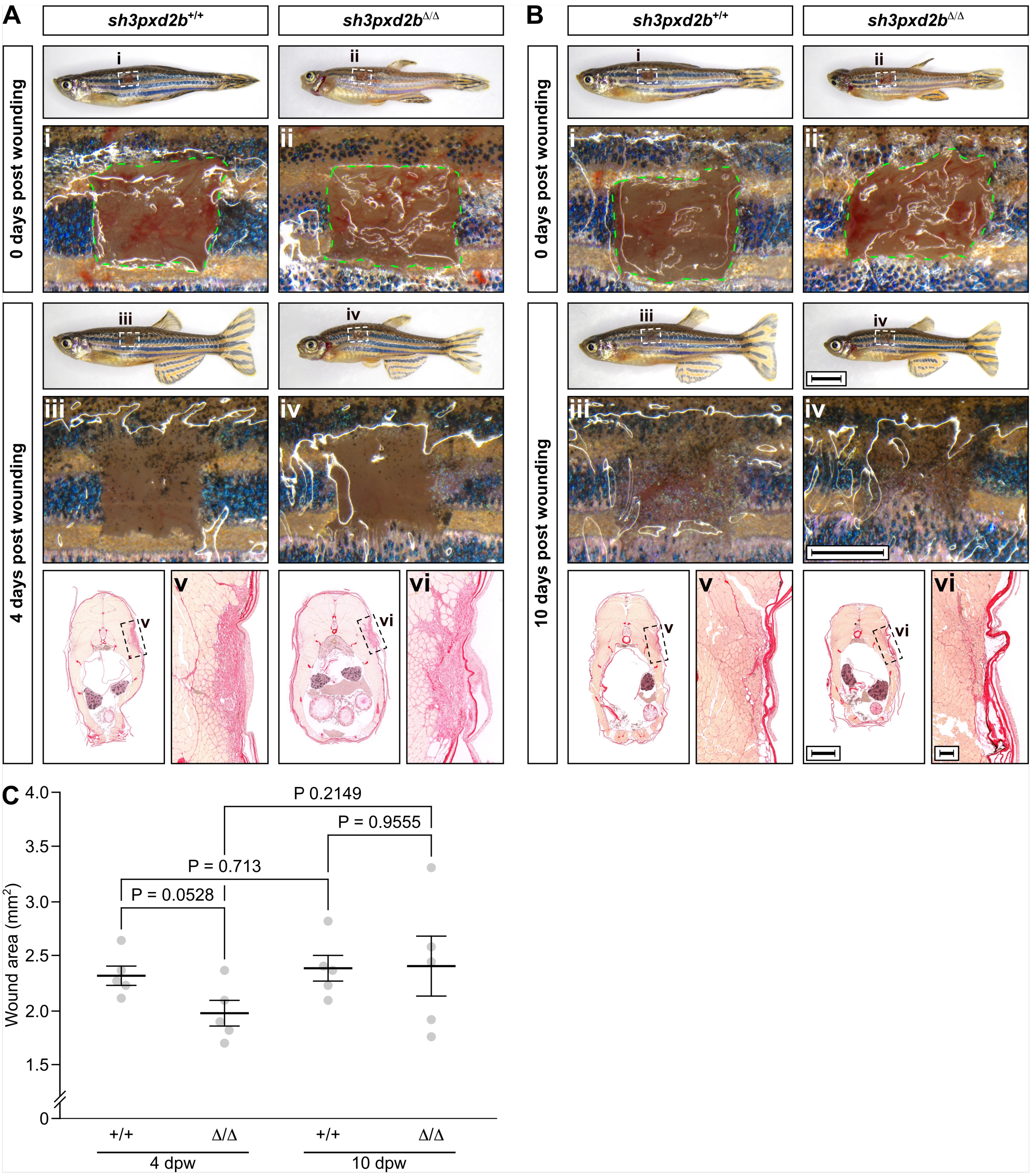

Figure S4 – Adult *sh3pxd2b*$^{\Delta/\Delta}$ zebrafish do not form excessive scar tissue when healing full-thickness cutaneous flank wounds. Representative images of 5-month-old *sh3pxd2b*$^{\Delta/\Delta}$ mutants and their *sh3pxd2b*$^{+/+}$ WT clutch mates, 4 or 10 days post flank wounding, selected from a total of five fish per genotype per time point. Gross anatomy images were taken with a Nikon SMZ25 stereomicroscope; sections were imaged with a Zeiss AxioImager Z.2 slide scanner. **A**, four days after a full-thickness cutaneous wound was inflicted on the left flank of adult mutants (i, wound edge demarcated by green dashed line) and their WT clutch mates (ii), both genotypes closed the wound and responed with migration of melanophores into the wound area (iii, iv) and (sub)cutaneous collagen accumulation (v, vi). **B**, ten days after full-thickness wounding, melanophores and iridophores have migrated into the wounded area in both WT fish (iii) and mutants (iv). In both genotypes, the (sub)cutaneous collagen deposition underlying the wounded area is comparable (v, vi) and similar to the surrounding unwounded area. Scale bar equals 5 mm in fish overview, 1 mm in wound close-up and section overview, and 100 µm in section close-up. **C**, wound area measurements of the five fish per genotype per time point. The average size of the inflicted flank wounds did not significantly differ between genotypes (P = 0.0528 for 4 dpw, P = 0.9555 for 10 dpw) or time points within genotypes (P = 0.713 for WT (+/+), P = 0.2149 for mutant (Δ/Δ), assessed by two-sampled, non-pooled, two-tailed Student's t-test). Error bars represent standard error of means of biological replicates.

Biology Open • Supplementary information

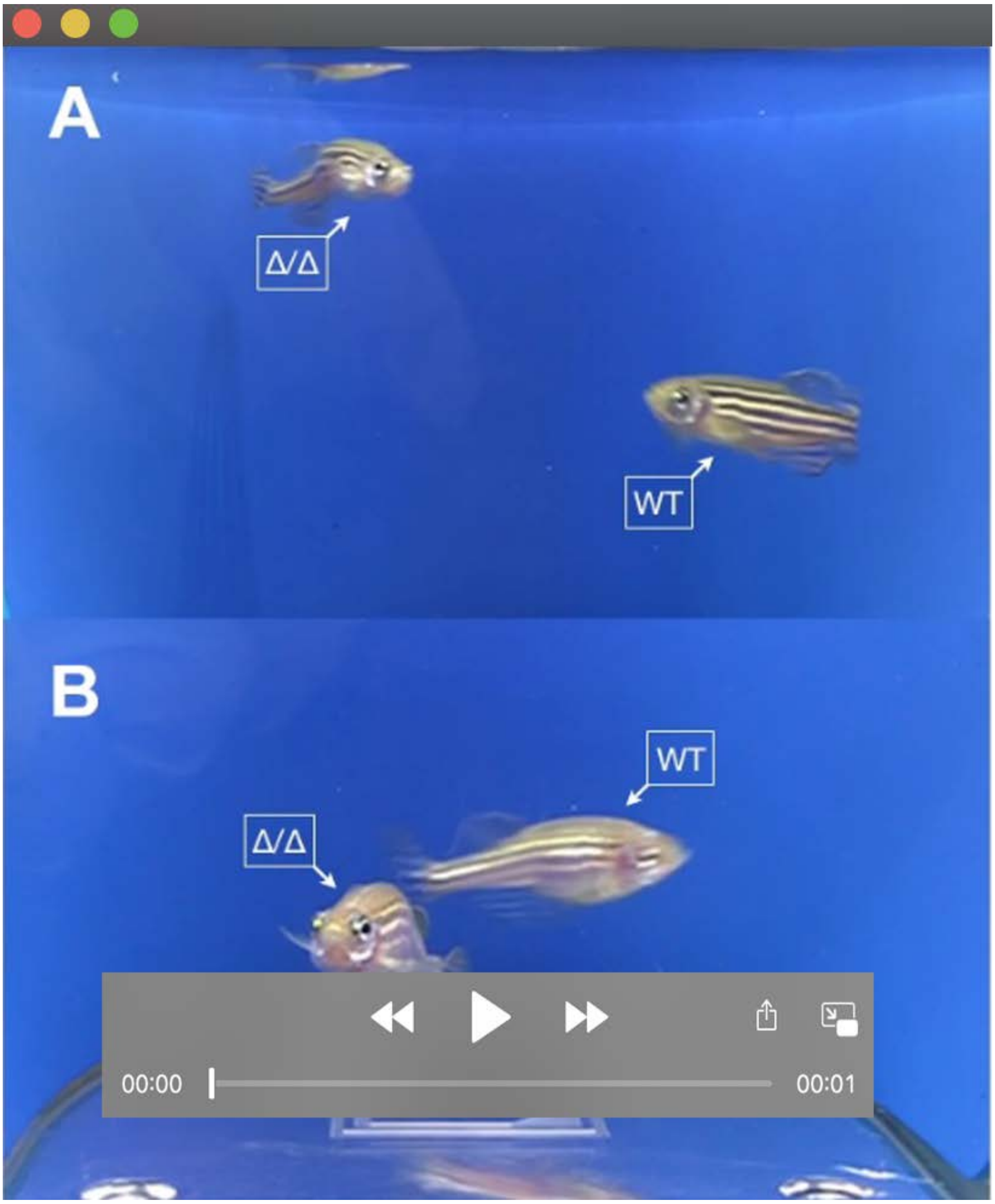

Movie 1 – Adult *sh3pxd2b*$^{\Delta/\Delta}$ fish develop fibrosis in their fins, affecting their swimming pattern. One-year-old *sh3pxd2b*$^{\Delta/\Delta}$ males (A) and females (B) develop gradually worsening fibrosis in the pectoral, dorsal, and anal fins, and the flank between the latter two fins, impairing movement of the affected fins.
